# Medial temporal lobe atrophy is associated with age and pathologies, especially small vessel disease

**DOI:** 10.1038/s41598-025-33602-6

**Published:** 2026-01-29

**Authors:** Danielle van Westen, Erik Stomrud, Luigi Lorenzini, Sebastian Palmqvist, Frederik Barkhof, Oskar Hansson, Nicola Spotorno

**Affiliations:** 1 Diagnostic Radiology, Department of Clinical Sciences Lund, Lund University, Klinikgatan 28, 222 42 Lund, Sweden; 2 Image and Function, Skåne University Hospital, 22242 Lund, Sweden; 3 Clinical Memory Research Unit, Department of Clinical Sciences Malmö, Lund University, 22242 Lund, Sweden; 4 Memory Clinic, Skåne University Hospital, Malmö, Sweden; 5 Department of Radiology and NuclearMedicine, De Boelelaan 1117, 1081 HV Amsterdam, The Netherlands; 6 Institutes of Neurology and Healthcare Engineering, University College London, London, UK

**Keywords:** Medial temporal lobe atrophy, Visual assessment, White matter hyperintensities, Amyloid pathology, Tau pathology, Alzheimer's disease, Neurovascular disorders

## Abstract

Visual assessment of medial temporal lobe atrophy (MTA) in the clinical workup of cognitive impairment is traditionally corrected for age since MTA increases with age. In addition, common pathologies in the elderly such as amyloid, tau, alpha-synuclein and TDP-43 accumulation as well as white matter hyperintensities representing small vessel disease may affect the association between MTA and age. We investigated this in 949 cognitively unimpaired (CU) and 854 cognitively impaired (CI) individuals focusing on amyloid, tau and alpha-synuclein that at present can be measured in vivo in plasma, CSF or using PET. MTA was associated with age also when these aforementioned pathologies were accounted for. WMH was the strongest and most consistent predictor and mediated 32–41% of the association between age and MTA. Secondly, an age-independent cut-off for distinguishing between Aβ- CU and Aβ + CI was derived from 195 CU participants with low levels of pathology. Accuracy, sensitivity and specificity were comparable for our age-independent and previously published age-adjusted cut-offs. In summary, age and WMH emerged as the most prominent factors associated with MTA. Our age-independent cut-off for MTA performed in line with the best performing age-adjusted cut-off, suggesting our more parsimonious proposal could be useful in a clinical setting.

## Introduction

Medial temporal lobe atrophy (MTA) is a morphological hallmark for Alzheimer’s disease (AD)^1^. In the early nineties, an ordinal rating scale ranging from 0 (no atrophy) to 4 (end-stage atrophy) was introduced for visual assessment of MTA from Magnetic Resonance Imaging (MRI) images as part of the clinical workup of people under investigation for cognitive impairment^2^. The Scheltens scale for visual assessment of MTA was designed to differentiate people with AD from cognitively unimpaired (CU) using an age-adjusted cut-off, since MTA increases with age also in CU individuals^3,4^. The original paper suggested a cut-off of ≥ 1 below 75 years and ≥ 1.5 above 75 years, average MTA score of the right and left side. Slightly modified, this cut-off is often used in clinical practice with MTA grade < 2 considered normal under the age of 75 and MTA grade < 3 over the age of 75^2^. It has since been tested and revised. For example, a study by Claus and colleagues, reported cut-offs of < 65, 65–74, 75–84 and ≥ 85 years of ≥ 1.0, ≥ 1.5, ≥ 2.0 and ≥ 2.0 for distinguishing patients with AD from participants with subjective memory impairment^5^.

In addition to age, MTA is affected by cerebral pathologies. For example, the presence and progression of white matter hyperintensities (WMH), the most common manifestation of cerebral small vessel disease (SVD), were associated with progression of MTA in AD^6^. Also, WMH burden was positively associated with hippocampal atrophy rate in controls and individuals with mild cognitive deficit (MCI)^4^. The MTA-score correlated with amyloid beta (Aβ) 42 in the cerebrospinal fluid (CSF) in subjects with MCI. In CU people, higher cerebral Aβ42 load estimated using PET was associated with increased longitudinal atrophy in the medial temporal lobe, even when accounting for age^7,8^. Increased Aβ42 and tau were both associated with atrophy of the medial temporal lobe (MTL) and a mediation analysis revealed that the relationship with regional atrophy was explained by tau accumulation^9^. These effects of neurodegenerative disease as well as SVD on MTA, raise the question to what extent they affect the abovementioned cut-offs and whether they influence the relationship between MTA and age. Of note, several papers reporting cut-offs for differentiation between CU and AD were based on a clinical diagnosis of AD and not on AD biomarkers^2,4,10,11^.

Therefore, we firstly examined the association between MTA and age while correcting for pathologies that can be measured in vivo at present, namely amyloid, tau and alpha-synuclein accumulation as well as WMH. In addition, we performed a mediation analysis to investigate to what extent these pathologies affect the association between age and MTA. Analysis was first performed in a derivation cohort and then replicated in a validation cohort that both include CU and cognitively impaired (CI) participants.

Secondly, we investigated if a simple, age-independent cut-off could distinguish between Aβ42-negative CU and Aβ42-positive CI as accurately as the clinically used age-adjusted cut-off and the cut-off proposed by Claus et al.^5^. The age-independent cut-off was derived from a reference group that consisted of CU people from the derivation cohort without evidence of Aβ42, tau or alpha-synuclein pathology and with low levels of WMH.

## Results

Demographic data for both cohorts are presented in Table 1. In the derivation cohort, sex distribution and education were similar in CU and CI participants. In the validation cohort, age was similar in CU and CI. In both cohorts, CI participants were older, were more often Aβ42 and SAA α-syn positive and had higher plasma p-tau217 levels, WMH volume and MTA score.

**Table 1:** Demographic data for the derivation and validation cohorts.

	Derivation cohort		Validation cohort	
Variable	CU (N = 600)	CI (N = 691)	CU (N = 358)	CI (N = 163)
Age, years	68.3 (10.3)	72.4 (7.5)*	72.6 (5.6)	71.9 (5.5)
Sex, female (%)	321 (54%)	310 (45%)	219 (61%)	72 (44%)*
Education, years	13 (4)	12 (4)	12 (4)	11 (3)*
MMSE, points	29 (1)	24 (4)*	29 (1)	27 (2)*
MTA, points	0.6 (0.7)	1.5 (1.0)*	0.8 (0.5)	1.3 (0.7)*
WMH (mm^3^)	6190 (5568)	9260 (6786)*	4924 (4502)	7646 (5556)*
Aβ42-positivity, n (%)	170 (28)	455 (66)*	109 (31)	104 (64)*
Plasma p-tau217 (pg ml^-1^)	0.21 (0.13)	0.45 (0.38)*	0.21 (0.12)	0.32 (0.21)*
CSF α-syn SAA positivity (LB pathology), n (%)	67 (11%)	180 (26%)*	36 (10%)	34 (21%)*

* denotes significant difference between cognitively unimpaired (CU) and cognitively impaired (CI) participants in each cohort (p < 0.05); MMSE = Mini Mental State Examination; MTA = Medial temporal lobe atrophy; WMH = White matter hyperintensities (volume from MRI); Aβ = amyloid beta 42; α-syn SAA = Seed amplification assay for detecting α-syn pathology; LB = Lewy body pathology. Of note, the CI group in the derivation cohort comprises both participants with MCI and AD dementia, and in the validation cohort participants with MCI only.

We created a reference group from the derivation cohort. This reference group comprised all CU participants without significant Aβ42, tau and alpha-synuclein pathology as well as a low level of WMH as proxy for SVD, see the Methods section for detailed description. These criteria lead to the inclusion of 195 participants (Table 2). As may be expected, this reference group was younger; by definition all participants were Aβ, tau and SAA α-syn negative and their mean WMH volume was low.

**Table 2:** Demographic data for the reference group.

Variable	N = 195
Age, years	60 (8)
Sex, female (%)	115 (59%)
Education, years	13 (3)
MMSE, points	29 (1)
MTA, points	0.2 (0.4)
WMH (mm^3^)	2666 (903)
Plasma P-tau217 (pg ml^-1^)	0.14 (0.05)
Aβ-PET SUVR	0.91 (0.04)
Tau-PET SUVR	1.13 (0.08)

The reference group consists of cognitively unimpaired participants without significant amyloid beta (Aβ) 42, tau and α-synuclein pathology as well as a low levels of WMH from the derivation cohort. Aβ-PET SUVR values were extracted from a composite neocortical ROI while tau-PET SUVR values were averaged across regions encompassing Braak stages I-IV. WMH were also assessed according to the Fazekas scale with average was 0.82, consistent with low level WMH.

### Association between MTA, age and markers of pathologies

The association between MTA and age was significant in the derivation and validation cohorts as well as in the CU and CI groups of each cohort (Table 3, a). In the reference group, that as mentioned consists of individuals from the derivation cohort, there was also an association between MTA and age, however much weaker than in the total group (β = 0.12, p = 0.001).

**Table 3:** Associations between MTA, age and pathologies.

	*Derivation cohort*			*Validation cohort*		
	*Whole*	*CU*	*CI*	*Whole*	*CU*	*CI*
*a. Linear regression model testing the association between MTA and age*						
Age	β = 0.45*	β = 0.34*	β = 0.42*	β = 0.27*	β = 0.29*	β = 0.29*
*b. Linear regression models including MTA, age and markers of pathology*						
Age	β = 0.23*	β = 0.21*	β = 0.28*	β = 0.15*	β = 0.20*	β = -0.196n.s
Plasma p-tau217	β = 0.23*	β = 0.13	β = 0.15*	**β = 0.12** *p* = 0.003	β = 0.12	β = -0.001
SAA α-syn	β = 0.09	β = 0.17	*β* = *-0.08*	*β* = *-0.08*	*β* = *-0.08*	β = -0.32
WMH	β = 0.32*	β = 0.22*	β = 0.33*	β = 0.34*	β = 0.24*	β = 0.31*
*c. Linear regression models with MTA, age and markers of pathology measured with PET, derivation cohort only*						
Age	β = 0.21*	β = 0.20*	β = 0.32*			
Amyloid PET	β = 0.06	β = 0.07	*β* = *-0.03*			
Tau PET	β = 0.17*	*β* = *-0.01*	β = 0.20*			
SAA α-syn	β = 0.13	**β = 0.30** p = 0.004	*β* = *-0.08*			
WMH	β = 0.30*	β = 0.22*	β = 0.37*			

Linear regression models testing (a) the association between MTA and age, (b) testing the association between MTA, age and markers of pathologies, with tau accumulation estimated in plasma and (c) testing the association between MTA, age and markers of pathologies, with amyloid and tau accumulation estimated using PET. For the latter only data from the derivation cohort were available. Values denote betas and associations that remain significant after accounting for multiple comparisons are denoted in bold; * denotes *p* < 0.001. WMH was the most consistent predictor, while the association with age was non-significant in the CI of the validation cohort, and with p-tau217 was non-significant in the CU participants of both cohorts. SAA alpha-syn was not a significant predictor in any analysis except in the derivation cohort and only when amyloid and tau pathology were estimated using PET.

We then investigated whether MTA was associated with age also when neurodegenerative diseases and SVD were corrected for using linear regression models (Table 3, b). In the derivation cohort, we found that the association between age and MTA was still significant. Furthermore, MTA was associated with WMH volume and with plasma levels of p-tau217 in the whole cohort and the CI group but not in the CU group. MTA was not associated with the proportion of SAA α-syn positive participants in any group.

Repeated analysis using Aβ- and tau-PET for estimation of AD pathology gave similar results with a significant association between age and MTA in the whole cohort, the CU and the CI group (Table 3, c). Again MTA was associated with WML load in all groups. Tau-PET uptake in a temporal meta-ROI was associated with MTA in the whole cohort and in the CI group but not in the CU group. Aβ-PET uptake, instead, was not associated with MTA. MTA was associated with the proportion of SAA α-syn positive participants in the CU group but not in the other analyses.

In the validation cohort, MTA was also associated with age when including markers of pathologies in the model, although in the CI group the association was only marginally significant after accounting for multiple comparisons. MTA was associated with WMH volume in all groups also in the validation cohort while the association with p-tau217 levels was significant only in the whole cohort. No association between MTA and SAA α-syn was found.

### Mediation analysis including MTA, age and WMH

**Fig. 1:**
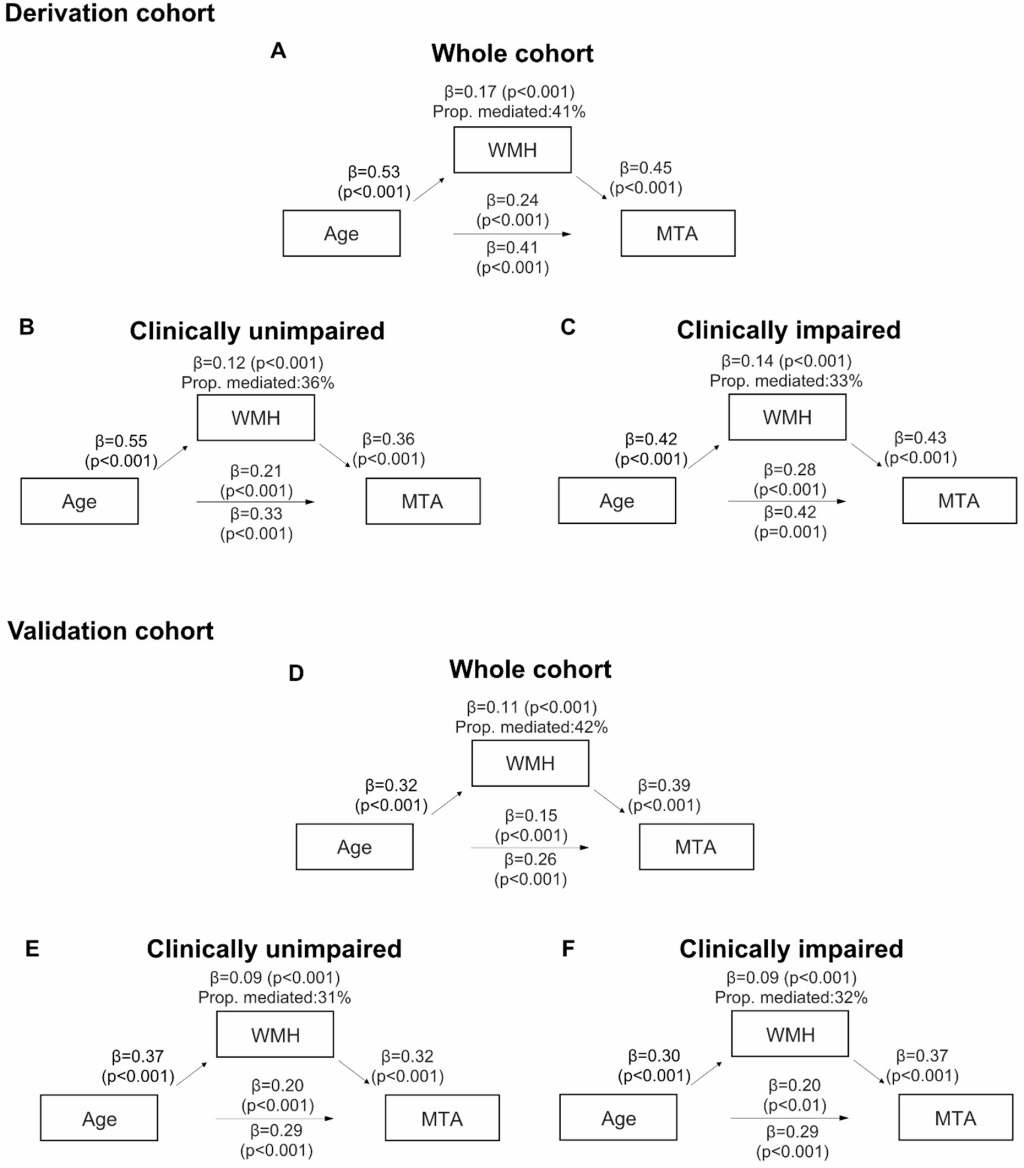
Mediation analysis of the effect of WMH on the association between MTA and age in both cohorts. Panels A and D show the whole, panels B and E the CU and panels B and E the CI participants. In the derivation cohort, WMH mediated 40% of the association between age and MTA in the whole cohort, 36% in the CU and 30% in the CI group. In the validation cohort, WMH mediated 42% of the association between age and MTA in the whole cohort and 31% in the CU and 32% in the CI group.

Since WMH was the most consistent and significant predictor of MTA aside from age, we performed mediation analysis to assess the extent to which WMH affect the association between age and MTA. In the derivation cohort, WMH emerged as a significant partial mediator which mediated 40% of the association between age and MTA in the whole cohort, 36% in the CU and 30% in the CI group. In the validation cohort, WMH mediated 42% of the association between age and MTA in the whole cohort and 31% in the CU and 32% in the CI group (Fig. 1).

### Performance of cut-offs

**Fig. 2:**
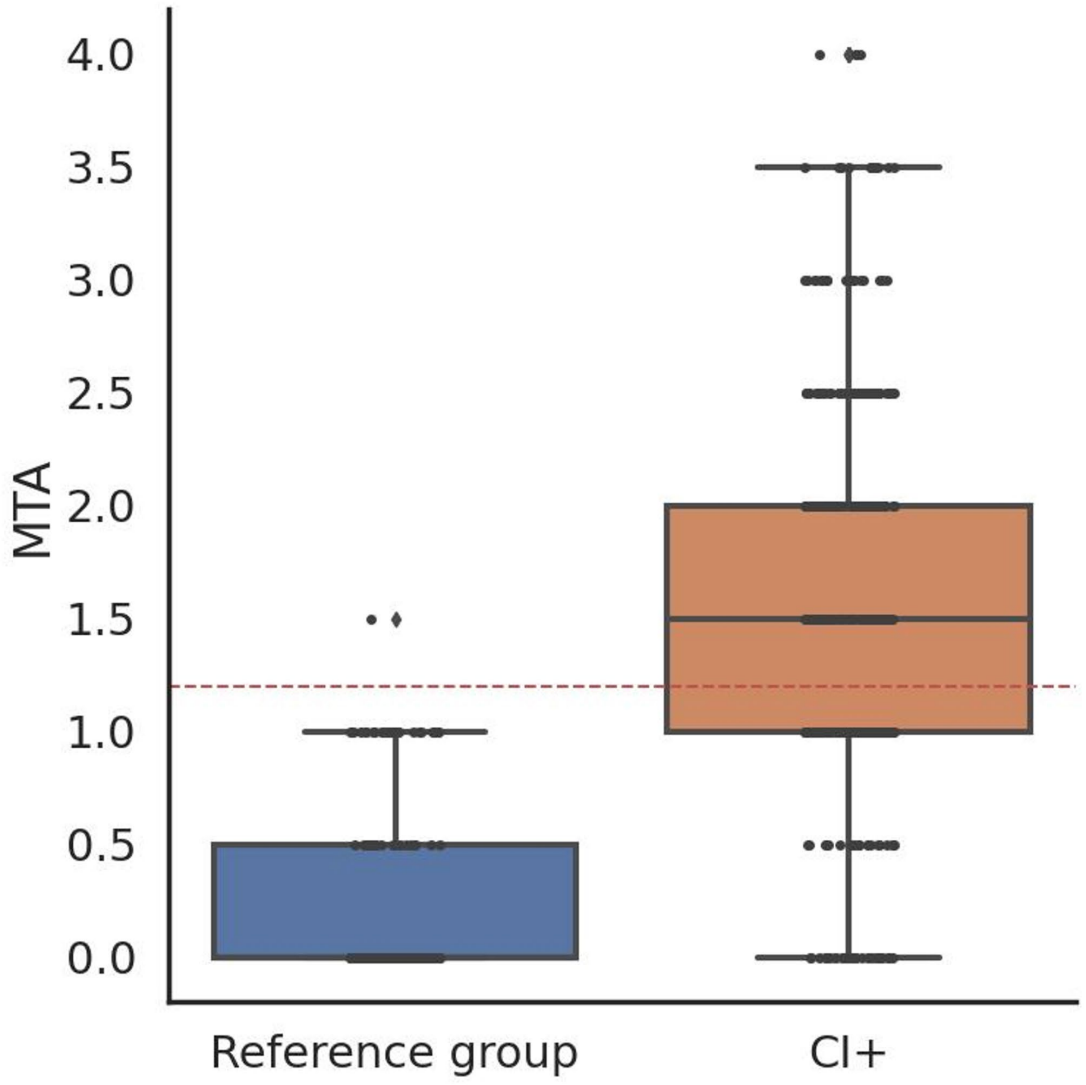
MTA in the reference group (boxplot to the left) and in the amyloid positive cognitively impaired (CI) participants from the derivation cohort (boxplot to the right), average of the left and right side. We created a reference group (n = 195) selecting partícipants from the cognitively unimpaired (CU) individuals in the derivation cohort that did not have any significant Aβ or tau, accumulation according to PET, nor alpha-synuclein pathology based on CSF seed amplification assay, and who had low levels of white matter hyperintensities (WMH). The average MTA in this reference group was 0.2, standard deviation 0.4. age-independent cut off was defined as 2.5 standard deviations being 1.16, the red dotted line in the boxplot. Few participants in the reference group had MTA grade 1.5 and none had MTA grade 2 or higher, while the range of the MTA grade in the CI group was 0–4 with mean 1.5.

The average MTA in the reference group was 0.2, standard deviation 0.4, resulting in a cut-off of MTA > 1, (defined as 2.5 times the standard deviation from the mean; MTA = 1.16) (Fig. 2).

We compared the performance of several cut-offs for discriminating Aβ negative CU participants from Aβ positive CI individuals, representing the AD continuum. Specifically we tested (1) the cut-off derived from the reference group defined as < 2.5 standard deviations of the average MTA in that group, resulting in MTA > 1; (2) the abovementioned cut-off used in clinical practice (MTA > 2 in individuals under 75 and MTA > 3 in individuals over 75 years); and (3) the cut-offs proposed by Claus et al^5^ (≥ 1.0, ≥ 1.5, ≥ 2.0 and ≥ 2.0 for the age ranges of: < 65, 65–74, 75–84 and ≥ 85 years, respectively).

**Fig. 3:**
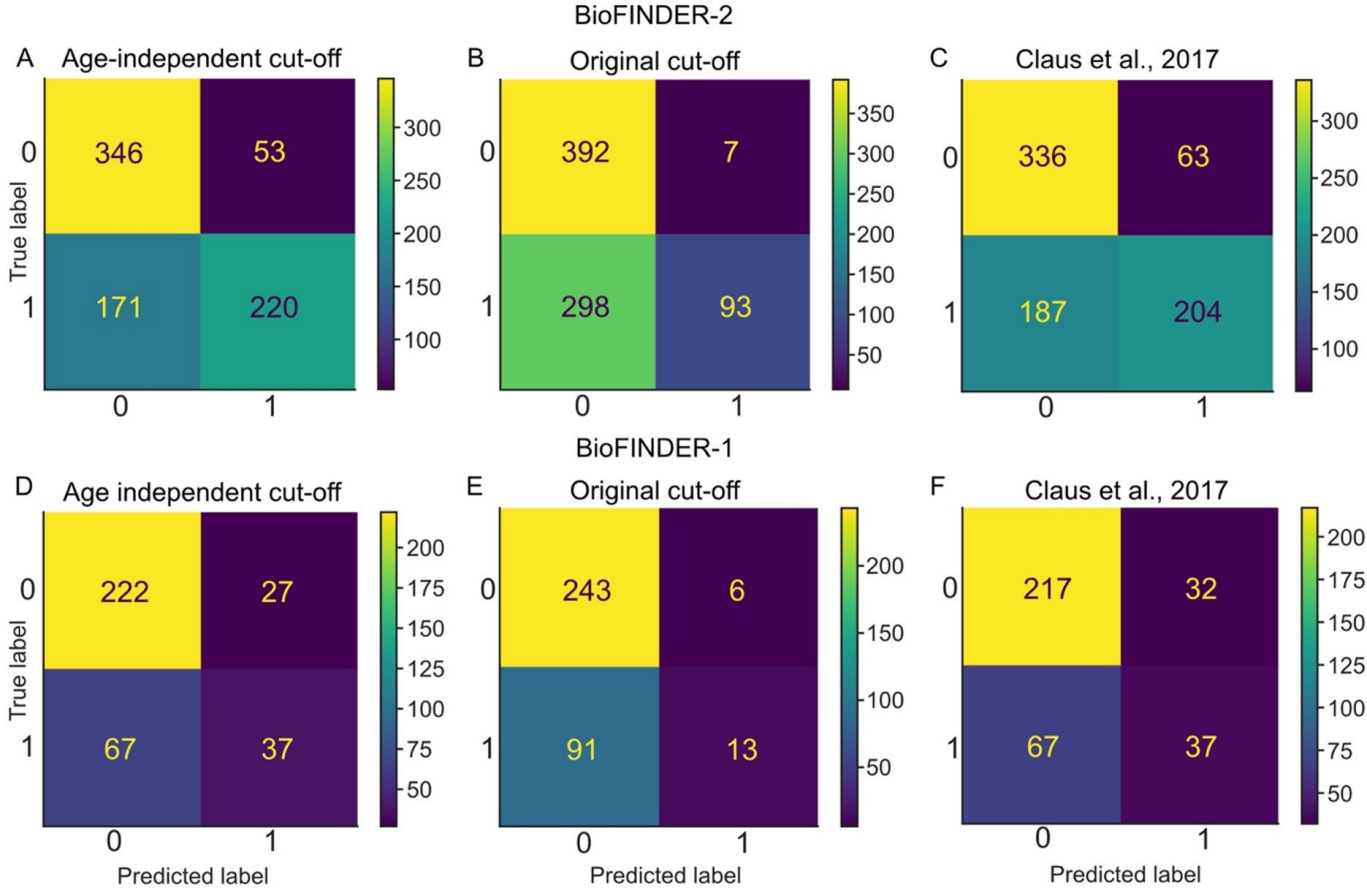
Confusion matrices for the age-independent cut-off and original cut-offs as well as the modified version according to Claus et al.^5^, in the derivation (panels **A**, **B** and **C**) and validation cohorts (panels **D**, **E** and **F**). See Table 4 for the accompanying estimates of accuracy, sensitivity and specificity.

In the derivation cohort, accuracy, sensitivity and specificity were 0.73, 0.88 and 0.57 for our proposed cut-off, respectively; 0.62, 0.99 and 0.24 for the clinically used cut-off and 0.69, 0.85 and 0.52 for the cut-off from Claus et al^5^ (Table 4; Fig. 3, panels A, B and C). In the validation cohort, accuracy, sensitivity and specificity were 0.77, 0.94 and 0.36 for our age-independent cut-off, respectively; 0.75, 0.99 and 0.14 for the clinically used cut-off and 0.76, 0.92 and 0.38 for the cut-off from Claus et al.^5^ (Table 4; Fig. 3, panels D, E and F). Thus, our proposed, age-independent cut-off showed slightly higher accuracy and a better balance between sensitivity and specificity compared to the performance of the clinically used age-adjusted cut-off.

**Table 4:** Performance of the cut-offs in the derivation and the validation cohorts.

	Derivation cohort			Validation cohort		
	Accuracy	Specificity	Sensitivity	Accuracy	Specificity	Sensitivity
Age-independent cut-off	0.73	0.88	0.57	0.77	0.94	0.36
Original cut-off^2^	0.62	0.99	0.24	0.75	0.99	0.14
Claus et al^5^.	0.69	0.85	0.52	0.76	0.92	0.38

Accuracy, sensitivity and specificity of the three cut-offs tested for distinguishing cognitively impaired from cognitively unimpaired people: (1) the age-independent cut-off, proposed in this paper, 2.5 standard deviations of the average MTA in the reference group (1.16, rounded to grade 1) (2) The original cut-off according to Scheltens^2^ with MTA grade > 1 being pathological in people < 75 years of age and grade > 2 abnormal in people > 75 years of age. (3) The cut-off according to Claus^5^ of < 65, 65–74, 75–84 and ≥ 85 years of ≥ 1.0, ≥ 1.5, ≥ 2.0 and ≥ 2.0 Overall, increased sensitivity is accompanied by decreased sensitivity while accuracy was comparable. The age-independent cut-off has a slightly better balance.

A sensitivity analysis excluding participants over 80 years of age showed almost identical results in both cohorts when the analysis was based on MTA being abnormal (> 1) in one hemisphere only (Table 5). Lastly, the consistency of the MTA rating was estimated based on additional ratings by two experienced raters in a subgroup of the validation cohort (N = 553). The intraclass correlation coefficient was 0.86, suggesting very good concordance between raters.

**Table 5:** Performance of the cut-offs in the derivation and the validation cohorts excluding participants over 80 years of age; abnormal MTA (> 1) is based on one hemisphere only.

	Derivation cohort			Validation cohort		
	Age ≤ 80					
	Accuracy	Specificity	Sensitivity	Accuracy	Specificity	Sensitivity
Age-independent cut-off	0.74	0.91	0.55	0.78	0.97	0.34
Original cut-off^2^	0.63	0.99	0.26	0.73	0.99	0.14
Claus et al^5^.	0.69	0.86	0.52	0.75	0.93	0.36

Accuracy, sensitivity and specificity of the three cut-offs tested for distinguishing cognitively impaired from cognitively unimpaired people, now excluding participants over 80 years of age: The age-independent cut-off, proposed in this paper, 2.5 standard deviations of the average MTA in the reference group (1.16, rounded to grade 1). (2) The original cut-off according to Scheltens^2^ with MTA grade > 1 being pathological in people < 75 years of age and grade > 2 abnormal in people > 75 years of age. (3) The cut-off according to Claus^5^ of < 65, 65–74, 75–84 and ≥ 85 years of ≥ 1.0, ≥ 1.5, ≥ 2.0 and ≥ 2.0 Overall, increased sensitivity is accompanied by decreased sensitivity while accuracy was comparable. The age-independent cut-off has a slightly better balance.

## Discussion

In summary, we firstly investigated the association between MTA, age and markers of neurodegenerative and WMH representing small vessel disease. Age and WMH were the variables most consistently associated with the MTA score. The mediation effect of WMH for the association between age and MTA was considerable, varying from 31–42% in both cohorts and subgroups. We then defined an age-independent cut-off for MTA of > 1 as the 2.5 standard deviation from the average MTA score in a reference group with low WMH as proxy for SVD and without evidence of AD and alpha-synuclein pathology. Our age-independent cut-off showed slightly higher accuracy and a better balance between sensitivity and specificity compared to the performance of the commonly used age adjusted cut-off.

In the clinic, visually assessed MTA is adjusted only for age, while often multiple cerebral pathologies are present, especially in older individuals. The positive association between MTA, age, tau, and WMH across cognitive status in both cohorts largely confirmed findings from previous studies. The absence of any association with Aβ-PET uptake is not surprising since accumulation of Aβ has been shown to start preferentially in regions connected to the posterior hippocampus, namely the precuneus, medial orbitofrontal, and posterior cingulate cortex^12^. Accumulation in the medial temporal area starts first in later clinical stages, when the mild cognitive impairment stage of Alzheimer’s disease is reached. A previous study showed that MTA-score and lateral ventricle volume correlated with CSF Aβ42 in individuals with MCI where half the cohort had pathological levels of CSF Aβ42^7,13^. Thus, amyloid accumulation in the medial temporal lobe may not be expected to be associated with MTA in early stages of AD.

Regarding tau pathology, it is well known that neurofibrillary tangle accumulation occurs early in the medial temporal lobe in AD and is strongly correlated with neurodegeneration, atrophy and cognitive decline^14,15^. In agreement with previous studies, plasma p-tau217 and tau-PET were associated with MTA in the present work^16^.

WMH are the most common manifestation of SVD with prevalence increasing with age, ranging from approximately 10% to 20% in those approximately 60 years old to close to 100% in those older than 90 years^17^. In concordance, WMH in our cohorts increased from the reference group to CU to CI individuals. Presence and progression of WMH are reportedly associated with progression of MTA in AD underscoring the importance of WMH in relation to MTA, as found in the present study^6^. WMH burden was positively associated with hippocampal atrophy rate in controls and individuals with mild cognitive deficit (MCI)^3,4^. However, the focus of the present study was the medial temporal lobe, an area that comprises not only the hippocampus but also white matter in the parahippocampal gyrus and adjacent subcortical tissue. WMH are commonly abundant in the periventricular area in the frontal and parietal lobes and usually less so in the temporal lobe. It might be speculated that periventricular parietal WMH affect white matter tracts running through the parietal and temporal lobes, such as the inferior fronto-occipital fasciculus and, following this pathway, impact MTA^18^.

WMH exerted a considerable mediation effect on the association between age and MTA. In AD research, WMH have usually been considered to reflect vascular lesions, as such contributing to cognitive impairment and dementia. Such lesions have been strongly linked to vascular risk factors like chronic hypertension, diabetes, and smoking^19,20^. Therefore, these results may highlight a possible pathway that leads from modifiable risk factor to medial temporal lobe degeneration. However, the heterogeneous pathophysiology of WMH has been suggested to involve non-vascular mechanisms such as AD-related neuroinflammation and neurodegeneration and thus part of WMH may be secondary to AD-related processes^21^. In addition, hypoperfusion has been shown to promote tau pathology in an animal model and in humans increased white matter hyperintensity volume was associated with higher plasma total tau concentration, particularly among those diagnosed clinically with Alzheimer’s disease^22^. Postmortem neuropathology in an older cohort showed that increased arteriolosclerosis was associated with a higher burden of tau-tangles specifically in the neocortex but not with β-amyloid potentially suggesting that SVD promotes tau pathology and the development of AD^23^.

The main limitation in the present study is that we could include only presently available biomarkers for neurodegenerative disease and thus the effect of TDP-43 on the association between MTA and age remains to be determined. Its presence maybe considerable, especially in advanced age. For example, in a community-based cohort at death (mean age 89.7 years), TDP-43 was present in 17.3% of cases with amnestic ‘AD-type’ dementia, AD neuropathologic change in 39.4% and all type cerebrovascular disease in 24.8%^24^.However, only a small percentage occurs before the age of 75^25^. In addition, tau and TDP-43 pathology are positively associated and contribute to MTA after correcting for other neuropathologies^24^. A potential synergistic effect on atrophy and the relative timing remains unclear.

In general, differences in results from our derivation and validation cohorts should at least partly be due to their intrinsic dissimilarities, with the validation cohort including controls and people with MCI and AD and the derivation cohort only including controls and people with MCI at baseline. This holds especially for the lower performance of our age-dependent cut-off in the validation cohort.

We studied the performance of an age-independent cut-off with MTA > 1 being abnormal, and found it similar to previously proposed age-dependent cut-offs regarding sensitivity and specificity (Table 4). Previous studies have recommended age-dependent cut-offs per decade, for example cut-offs of ≥ 1.5, ≥ 1.5, ≥ 2 and ≥ 2.5 for age ranges 45–64, 65–74, 75–84 and 85–94 years, respectively, for the diagnosis of AD compared to controls^11^. Although the performance is similar to our proposed cut-off, these age-dependent cut-offs per decade are less practical for clinical use. Moreover, differentiating between MTA grade 2 and 3 when the cut-off change at 75 years is used, can be difficult in practice and is more susceptible for intra- and inter-rater variability. Furthermore, with the clinical work-up of cognitive decline moving towards early detection AD pathology, AD cases with considerable medial temporal lobe atrophy will become less common. In the older age group specifically, the usefulness of age-dependent cut-offs for MTA in the older age group is limited due to reduced specificity or low sensitivity^5,26^. The original paper by Scheltens et al. on the MTA scoring system reported poor sensitivity for the diagnosis of AD in older patients and recommended its use in subjects younger than 75 years^2^. A postmortem MRI study in individuals over 85 years of age found MTA to be sensitive to primary degenerative hippocampal pathology in the very old, but not specific for Alzheimer-type pathology^27^.

In conclusion, we showed that the relationship between MTA and age remains significant, even when correcting for cerebrovascular pathology and neurodegenerative diseases where markers are available and that a considerable portion of this association is mediated by WMH. We evaluated the performance of an age-independent cut-off for MTA and found it performed similarly to the age-adjusted cut-offs. This age-independent cut-off would be more easy to use in clinical practice.

## Methods

### Participants

All participants were part of the BioFINDER-1 (NCT01208675; *n* = 521) and BioFINDER-2 (NCT03174938; *n* = 1291) studies described previously^28–30^. Both studies were approved by the Swedish Ethical Review Authority and the study was conducted in line with the Helsinki declaration. All participants provided written and oral informed consent.

BioFINDER-2 was the derivation and BioFINDER-1 the validation cohort. In the present study, only participants 50 years of age and above were included.

The derivation cohort comprised 600 clinically unimpaired (CU) participants and 691 cognitively impaired (CI), and the validation cohort 349 and 163, respectively. CU participants did not fulfill the criteria for MCI or dementia, as defined in the Diagnostic and Statistical Manual of Mental Disorders, 5th edition (DSM-5)^31^^.^ Thus this included cognitively healthy controls as well as participants with subjective cognitive decline who performed within normal ranges on a large cognitive test battery, that is, did not have MCI. The CI participants were diagnosed with MCI or dementia in the derivation cohort and with MCI in the validation cohort as per study design. MCI was classified as performing worse than − 1.5 × the standard deviation in at least one of the cognitive domains of memory, attention/executive, verbal or visuospatial function while not fulfilling the criteria for dementia. Dementia was classified according to the DSM-5 criteria for major neurocognitive disorders^31^. A clinical diagnosis of AD was based on the DSM-5 criteria for mild or major neurocognitive disorder due to AD and signs of Aβ positivity in agreement with the National Institute of Aging-Alzheimer’s Association and International Working Group criteria for AD^31,32^.

The purely biomarker-driven classification for AD pathology (Aβ and tau positivity, regardless of clinical syndrome) was used in the statistical analysis. In the derivation cohort Aβ status was defined based on Aβ-PET uptake in a neocortical composite region using a previously published cut-off of 1.033^33^. When Aβ-PET was not available, CSF Aβ42/40 ratio was used with a cut-off of 0.08 (note that preanalytical differences between cohorts resulted in different cut-offs)^34^. In the validation cohort, Aβ status was defined using a cut-off of 0.066 for the CSF Aβ42/40 ratio (Elecsys® Roche)^35,36^.

All participants or their legal representatives provided written informed consent. Ethical approval was given by the Regional Ethical Committee in Lund, Sweden.

### Reference group

A reference group was selected from the derivation cohort and consisted of CU participants without significant Aβ, tau and alpha-synuclein pathology as well as a low level of WMH. Aβ status was defined using the abovementioned cut-offs, 1.033 when based on Aβ-PET uptake in a neocortical composite region, or 0.08 when based on the CSF Aβ42/40 ratio (three cases). Tau positivity was defined based on tau-PET uptake from a temporal composite region using a previously published cut-off of 1.36^37^. A-syn negativity was defined based on the results of the α-Syn RT-QuIC analyses. Low level of WMH was defined as the first tertile of the distribution of WMH volume in the whole cohort; as a sanity check, WMH were assessed according to the Fazekas scale, the average was 0.82, consistent with low level WMH. These criteria led to the inclusion of 195 participants in the reference group (see Table 3).

### MRI, data acquisition and analysis

Participants in the derivation cohort underwent MRI on a Siemens MAGNETOM Prisma 3 T scanner (Siemens Healthineers, Erlangen, Germany) with a 64-channel head coil. A T1-weighted MPRAGE sequence (TR = 1900 ms TE = 2.54 ms, in-plane resolution = 1 × 1 mm^2^, slice thickness = 1 mm) and a T2-weighted FLAIR scan (TR = 5000 ms, TE = 393 ms, same resolution and FOV as for the MPRAGE) were acquired. Participants in the validation cohort underwent MRI on a 3 T MR scanner (Siemens Tim Trio 3 T, Siemens Healthineers, Erlangen, Germany), including T1-weighted (TR = 1950 ms, TE = 3.4 ms, in-plane resolution = 1 × 1 mm^2^, slice thickness = 1.2 mm) and axial T2 FLAIR imaging (27 slices, voxel size 0.7 × 0.7 × 5.2 mm3).

Image analysis included visual rating of MTA according to the Scheltens scale^2^ by the same rater (DvW) for both cohorts. In addition, two additional raters (FB and LL) visually assessed MTA in a subgroup from the derivation cohort (*n* = 553), blinded to sex, age as well as cognitive and biomarker status. WMH were quantified using the longitudinal Sequence Adaptive Multimodal SEGmentation (SAMSEG) tool from FreeSurfer (v7.2; https://surfer.nmr.mgh.harvard.edu/;v7.2) on the FLAIR images^38,39^. The resulting volume [mm^3^] for the whole brain was log transformed to adjust for skewness and corrected for the intracranial volume.

### Cerebrospinal fluid and plasma markers

Concentrations of CSF Aβ42 and Aβ40 were measured with the Roche Elecsys® assays^40^. Αlpha-synuclein (α-syn) RT-QuIC analyses were performed using an in vitro seeding amplification assay (SAA) generating a binary assessment of the presence of α-syn pathology (SAA α-syn positive or negative), as previously reported^41^. Plasma levels of p-tau217 were quantified using the Meso Scale Discovery platform using an assay developed by Eli Lilly.

### PET imaging and processing

In the derivation cohort, participants underwent [^18^F]-flutemetamol PET and [^18^F]-RO948 PET on Discovery MI scanners (GE healthcare). [^18^F]-Flutemetamol PET images were acquired 90 to 110 min after injection of 185 MBq 18F-flutemetamol while [^18^F]-RO948 PET images were acquired 70 to 90 min after injection of 370 MBq [^18^F]-RO948. Pre-processing and generation of standardized uptake value ratios (SUVR) maps were carried out as described in previous reports^12,26^. [^18^F]-Flutemetamol scans were normalized using the cerebellar cortex as reference region, while the inferior cerebellar gray matter was chosen as reference region for [^18^F]-RO948. By study design, participants enrolled when already at the dementia stage did not undergo [^18^F]-flutemetamol PET.

### Statistical analysis

Demographic characteristics were compared between groups using Student’s t-test or Chi-square analysis for continuous and binary variables respectively.

We defined MTA as abnormal based on the distribution of MTA values in the reference group. The cut-off was set at 2.5 standard deviations above the mean MTA of the reference group, resulting in MTA > 1 (see Results). Accuracy, sensitivity and specificity in distinguishing between Aβ- CU and Aβ + CI were computed based on our proposed, age-independent cut-off as well as the original age-adjusted MTA criteria (a cut-off of 75 years where MTA grade < 2 is considered normal under the age of 75 and MTA grade < 3) and the modified version proposed by Claus et al^5^. These analyses were first performed in the derivation cohort and repeated in the validation cohort.

The association between MTA and age in the reference group as well as in the two main cohorts was investigated using linear regression including sex as covariate. Then, in both main cohorts models were used to test the effect of the abovementioned co-pathologies on the association between MTA and age. MTA was used as outcome measure while age, plasma levels of p-tau217, α-Syn RT-QuIC, WMH volume and sex were included as predictors. These analyses were performed in the whole cohort as well as in the CU and CI groups separately. A sensitivity analysis in the derivation cohort comprised Aβ- and tau-PET as proxy of Aβ and tau pathology respectively instead of fluid markers. In this case, average neocortical Aβ-PET uptake was used as a proxy of Aβ burden and tau-PET uptake in a temporal meta-ROI encompassing regions corresponding to Braak stage I-IV was employed as a proxy of tau burden.

Finally, mediation analysis was used to assess the potential mediation effect on the association between MTA and age; the variables that emerged as the strongest and most consistent predictors of MTA across the multiple regression models were tested. Bonferroni correction for multiple comparisons was applied in all analyses.

## Data Availability

Anonymized data from BioFINDER will be shared on request from a qualified academic investigator for the sole purpose of replicating procedures and results presented in the article and as long as data transfer is in agreement with EU legislation on the general data protection regulation and decisions by the Swedish Ethical Review Authority and Region Skåne, which should be regulated in a material transfer agreement.

## References

[1] Braak, H. & Braak, E. Neuropathological stageing of Alzheimer-related changes. *Acta Neuropathol.* **82**, 239–259 (1991). 1759558 10.1007/BF00308809

[2] Scheltens, P. et al. Atrophy of medial temporal lobes on MRI in “probable” Alzheimer’s disease and normal ageing: Diagnostic value and neuropsychological correlates. *J. Neurol. Neurosurg. Psychiatry.* **55**, 967–972 (1992). 1431963 10.1136/jnnp.55.10.967 PMC1015202

[3] Berron, D. et al. Early stages of tau pathology and its associations with functional connectivity, atrophy and memory. *Brain* **144**, 2771–2783 (2021). 33725124 10.1093/brain/awab114 PMC8557349

[4] Fiford, C. M. et al. White matter hyperintensities are associated with disproportionate progressive hippocampal atrophy. *Hippocampus* **27**, 249–262 (2017). 27933676 10.1002/hipo.22690 PMC5324634

[5] Claus, J. J. et al. Practical use of visual medial temporal lobe atrophy cut-off scores in Alzheimer’s disease: Validation in a large memory clinic population. *Eur Radiol.* **27**, 3147–3155 (2017). 28083697 10.1007/s00330-016-4726-3 PMC5491609

[6] De Leeuw, F. E., Korf, E., Barkhof, F. & Scheltens, P. White matter lesions are associated with progression of medial temporal lobe atrophy in Alzheimer disease. *Stroke* **37**, 2248–2252 (2006). 16902173 10.1161/01.STR.0000236555.87674.e1

[7] Clerx, L. et al. Measurements of medial temporal lobe atrophy for prediction of Alzheimer’s disease in subjects with mild cognitive impairment. *Neurobiol. Aging.* **34**, 2003–2013 (2013). 23540941 10.1016/j.neurobiolaging.2013.02.002

[8] Nosheny, R. L. et al. Associations among amyloid status, age, and longitudinal regional brain atrophy in cognitively unimpaired older adults. *Neurobiol. Aging.* **82**, 110–119 (2019). 31437719 10.1016/j.neurobiolaging.2019.07.005 PMC7198229

[9] Marks, S. M., Lockhart, S. N., Baker, S. L. & Jagust, W. J. Tau and β-Amyloid are associated with medial temporal lobe structure, function, and memory encoding in normal aging. *J. Neurosci.* **37**, 3192–3201 (2017). 28213439 10.1523/JNEUROSCI.3769-16.2017 PMC5373113

[10] Molinder, A., Ziegelitz, D., Maier, S. E. & Eckerström, C. Validity and reliability of the medial temporal lobe atrophy scale in a memory clinic population. *BMC Neurol.* **21**, 289 (2021). 34301202 10.1186/s12883-021-02325-2 PMC8305846

[11] Ferreira, D. et al. Practical cut-offs for visual rating scales of medial temporal, frontal and posterior atrophy in Alzheimer’s disease and mild cognitive impairment. *J. Intern. Med.* **278**, 277–290 (2015). 25752192 10.1111/joim.12358

[12] Palmqvist, S. et al. Earliest accumulation of β-amyloid occurs within the default-mode network and concurrently affects brain connectivity. *Nat. Commun.* **31**, 1214 (2017). 10.1038/s41467-017-01150-x PMC5663717 29089479

[13] Hampel, H. et al. The amyloid-beta pathway in Alzheimer’s disease. *Mol. Psychiatry.* **26**, 5481–5503 (2021). 34456336 10.1038/s41380-021-01249-0 PMC8758495

[14] Braak, H. & Braak, E. Staging of Alzheimer’s disease-related neurofibrillary changes. *Neurobiol. Aging.* **16**, 271–278 (1995). 7566337 10.1016/0197-4580(95)00021-6

[15] Nelson, P. T. et al. Correlation of Alzheimer disease neuropathologic changes with cognitive status: A review of the literature. *J. Neuropathol. Exper. Neurol.* **71**, 362–381 (2012). 22487856 10.1097/NEN.0b013e31825018f7 PMC3560290

[16] Wisse, L. E. et al. Tau pathology mediates age effects on medial temporal lobe structure. *Neurobiol. Aging.* **109**, 135–144 (2022). 34740075 10.1016/j.neurobiolaging.2021.09.017 PMC8800343

[17] Smith, E. E. et al. Prevention of stroke in patients with silent cerebrovascular disease: A scientific statement for healthcare professionals from the American heart association/American stroke association. *Stroke* **48**, e44–e71 (2017). 27980126 10.1161/STR.0000000000000116

[18] Koohi, F., Harshfield, E. L. & Markus, H. S. Contribution of conventional cardiovascular risk factors to brain white matter hyperintensities. *J. Am. Heart Assoc.* **12**, e030676 (2023). 37421292 10.1161/JAHA.123.030676 PMC10382123

[19] Biesbroek, J. M. et al. Signature white matter hyperintensity locations associated with vascular risk factors derived from 15 653 individuals. *Stroke* **56**, 3047–3059 (2025). 40832713 10.1161/STROKEAHA.125.051159 PMC12447828

[20] Wu, Y., Sun, D., Wang, Y. & Wang, Y. Subcomponents and connectivity in the inferior frontal-occipital fasciculus revealed by diffusion spectrum imaging fiber tracking. *Front. Neuroanat.* **10**, 88 (2016). 27721745 10.3389/fnana.2016.00088 PMC5033953

[21] Garnier-Crussard, A. & Chételat, G. White matter hyperintensities in Alzheimer’s disease: Beyond (but not instead of) the vascular contribution. *Alzheimers Dement.* **19**, 4262–4263 (2023). 37437034 10.1002/alz.13372

[22] Laing, K. K. et al. Cerebrovascular disease promotes tau pathology in Alzheimer’s disease. *Brain Commun.* **2**, fcaa132 (2020). 33215083 10.1093/braincomms/fcaa132 PMC7660042

[23] Kapasi, A. et al. Association of small vessel disease with tau pathology. *Acta Neuropathol.* **143**, 349–362 (2022). 35044500 10.1007/s00401-021-02397-x PMC8858293

[24] Nelson, P. T., Schneider, J. A., Jicha, G. A., Duong, M. T. & Wolk, D. A. When Alzheimer’s is LATE: Why does it matter?. *Ann. Neurol.* **94**, 211–222 (2023). 37245084 10.1002/ana.26711 PMC10516307

[25] Nelson, P. T. et al. Limbic-predominant age-related TDP-43 encephalopathy (LATE): Consensus working group report. *Brain* **142**, 1503–1527 (2019). 31039256 10.1093/brain/awz099 PMC6536849

[26] Rhodius-Meester, H. F. M. et al. MRI visual ratings of brain atrophy and white matter hyperintensities across the spectrum of cognitive decline are differently affected by age and diagnosis. *Front. Aging Neurosci.* **9**, 117 (2017). 28536518 10.3389/fnagi.2017.00117 PMC5422528

[27] Barkhof, F. et al. The significance of medial temporal lobe atrophy: A postmortem MRI study in the very old. *Neurology* **69**, 1521–1527 (2007). 17923614 10.1212/01.wnl.0000277459.83543.99

[28] Palmqvist, S. et al. Discriminative accuracy of plasma phospho-tau217 for Alzheimer disease vs other neurodegenerative disorders. *JAMA* **324**, 772–781 (2020). 32722745 10.1001/jama.2020.12134 PMC7388060

[29] Palmqvist, S. et al. Performance of fully automated plasma assays as screening tests for Alzheimer disease-related beta-amyloid status. *JAMA Neurol.* **76**, 1060–1069 (2019). 31233127 10.1001/jamaneurol.2019.1632 PMC6593637

[30] Palmqvist, S. et al. Prediction of future Alzheimer’s disease dementia using plasma phospho-tau combined with other accessible measures. *Nat. Med.* **27**, 1034–1042 (2021). 34031605 10.1038/s41591-021-01348-z

[31] American Psychiatric Association. Diagnostic and Statistical Manual of Mental Disorders, Fifth Edition (DSM-5) 607–608 (American Psychiatric Publishing, 2013).

[32] Dubois, B. et al. Clinical diagnosis of Alzheimer’s disease: Recommendations of the International Working Group. *Lancet Neurol.* **20**, 484–496 (2021). 33933186 10.1016/S1474-4422(21)00066-1 PMC8339877

[33] Brum, W. S. et al. A two-step workflow based on plasma p-tau217 to screen for amyloid β positivity with further confirmatory testing only in uncertain cases. *Nat. Aging.* **3**, 1079–1090 (2023). 37653254 10.1038/s43587-023-00471-5 PMC10501903

[34] Pichet Binette, A. et al. Amyloid-associated increases in soluble tau relate to tau aggregation rates and cognitive decline in early Alzheimer’s disease. *Nat. Commun.* **13**, 6635 (2022). 36333294 10.1038/s41467-022-34129-4 PMC9636262

[35] Cullen, N. C. et al. Plasma biomarkers of Alzheimer’s disease improve prediction of cognitive decline in cognitively unimpaired elderly populations. *Nat. Commun.* **12**, 3555 (2021). 34117234 10.1038/s41467-021-23746-0 PMC8196018

[36] Palmqvist, S. et al. An accurate fully automated panel of plasma biomarkers for Alzheimer’s disease. *Alzheimers Dement.* **19**, 1204–1215 (2023). 35950735 10.1002/alz.12751 PMC9918613

[37] Leuzy, A. et al. Diagnostic performance of RO948 F 18 tau positron emission tomography in the differentiation of Alzheimer disease from other neurodegenerative disorders. *JAMA Neurol.* **77**, 955–965 (2020). 32391858 10.1001/jamaneurol.2020.0989 PMC7215644

[38] Puonti, O., Iglesias, J. E. & Van Leemput, K. Fast and sequence-adaptive whole-brain segmentation using parametric Bayesian modeling. *Neuroimage* **143**, 235–249 (2016). 27612647 10.1016/j.neuroimage.2016.09.011 PMC8117726

[39] Cerri, S. et al. A contrast-adaptive method for simultaneous whole-brain and lesion segmentation in multiple sclerosis. *Neuroimage* **225**, 117471 (2021). 33099007 10.1016/j.neuroimage.2020.117471 PMC7856304

[40] Bittner, T. et al. Technical performance of a novel, fully automated electrochemiluminescence immunoassay for the quantitation of β-amyloid (1–42) in human cerebrospinal fluid. *Alzheimers Dement.* **12**, 517–1526 (2016). 26555316 10.1016/j.jalz.2015.09.009

[41] Bellomo, G. et al. alpha-Synuclein seed amplification assays for diagnosing synucleinopathies: The way forward. *Neurology* **99**, 195–205 (2022). 35914941 10.1212/WNL.0000000000200878

